# Analysis of a Standardized Technique for Laparoscopic Cuff Closure following 1924 Total Laparoscopic Hysterectomies

**DOI:** 10.1155/2016/1372685

**Published:** 2016-08-04

**Authors:** Katherine A. O'Hanlan, Pamela L. Emeney, Alfred Peters, Margaret S. Sten, Stacey P. McCutcheon, Danielle M. Struck, Joseph K. Hoang

**Affiliations:** ^1^Laparoscopic Institute for Gynecology and Oncology, 4370 Alpine Road, Portola Valley, CA 94028, USA; ^2^University of California at San Francisco-Fresno, 155 North Fresno Street, Fresno, CA 93701, USA

## Abstract

*Objective*. To review the vaginal cuff complications from a large series of total laparoscopic hysterectomies in which the laparoscopic culdotomy closure was highly standardized.* Methods*. Retrospective cohort study (Canadian Task Force Classification II-3) of consecutive total and radical laparoscopic hysterectomy patients with all culdotomy closures performed laparoscopically was conducted using three guidelines: placement of all sutures 5 mm deep from the vaginal edge with a 5 mm interval, incorporation of the uterosacral ligaments with the pubocervical fascia at each angle, and, whenever possible, suturing the bladder peritoneum over the vaginal cuff edge utilizing two suture types of comparable tensile strength. Four outcomes are reviewed: dehiscence, bleeding, infection, and adhesions.* Results*. Of 1924 patients undergoing total laparoscopic hysterectomy, 44 patients (2.29%) experienced a vaginal cuff complication, with 19 (0.99%) requiring reoperation. Five patients (0.26%) had dehiscence after sexual penetration on days 30–83, with 3 requiring reoperation. Thirteen patients (0.68%) developed bleeding, with 9 (0.47%) requiring reoperation. Twenty-three (1.20%) patients developed infections, with 4 (0.21%) requiring reoperation. Three patients (0.16%) developed obstructive small bowel adhesions to the cuff requiring laparoscopic lysis.* Conclusion*. A running 5 mm deep × 5 mm apart culdotomy closure that incorporates the uterosacral ligaments with the pubocervical fascia, with reperitonealization when possible, appears to be associated with few postoperative vaginal cuff complications.

## 1. Introduction

Postlaparoscopic hysterectomy vaginal cuff complications, such as dehiscence, bleeding, infection, and adhesions, are infrequent but can potentially lead to more serious problems including acute anemia, evisceration, bowel injury, peritonitis, sepsis, and reoperation. A recent review of 57 cohort studies of one type of complication, cuff dehiscence, after laparoscopic hysterectomy found that transvaginal closure of the vaginal cuff was associated with the lowest dehiscence rate as compared to laparoscopic and robotic cuff closures [1]. However, variations in vaginal anatomy associated with nulliparity, obesity, and senescent vaginal constrictive changes can make transvaginal culdotomy closure difficult or impossible, underscoring the need for an effective laparoscopic approach for culdotomy closure. Additionally, other notable vaginal cuff complications, such as bleeding, infection, and postoperative adhesions, require further investigation with regard to the closure technique. Surgeon experience may also play a role and may affect the reliability of the closure.

The objective of this retrospective report and video is to review the association of the surgeon's experience and closure guidelines that include a consistent 5 mm interval with a 5mm depth from the vaginal cuff edge, incorporation of the uterosacral ligaments with the pubocervical fascia at each angle, and closure of the peritoneum over the vaginal cuff whenever possible, with the four major categories of complications: dehiscence, bleeding, infections, and adhesions.

## 2. Materials and Methods

With Investigational Review Board approval from Sequoia Hospital in Redwood City, CA, data for every patient undergoing total laparoscopic hysterectomy and concomitant procedures from September 1, 1996, to April 7, 2015, was abstracted from hospital and office files, anonymized, and stored on an excel spreadsheet. The patient's history and physical examination, operative report, and hospital record were reviewed to obtain age, BMI, estimated blood loss, procedure performed, and surgical duration. For this report, postoperative vaginal complications were defined as any vaginal apex or cuff-related complication, including dehiscence, bleeding, infection, or adhesions occurring up to 90 days after surgery [2]. Pelvic cellulitis was included as an infectious complication and was defined as a vague abdominal pain or the sensation of pelvic fullness, with apical vaginal induration, tenderness to palpation, and edema in the absence of abscess or peritoneal signs [3].

Surgeon experience was assessed by compiling the complications in each sequential segment of 200 cases, with 10 segments, in the series of 1924 patients.

In this practice, all patients needing hysterectomy for benign or malignant indications were scheduled for a laparoscopic approach unless they had radiologically documented metastatic disease or prior operative records documenting severe abdominal/intestinal adhesions and were excluded from this study. Every patient signed that she had read a 13-page document called “Pre-operative planning and recovery information” (available at http://www.ohanlan.com/pdf/patient_ed_planning_surgery_pdf/preoperative_instructions_and_recovery.pdf) which details the anticipated pre- and postoperative hysterectomy experience and the possible complications in this practice and provides detailed posthospital instructions. In all patients, single abdominal-vulvar-vaginal and perineal preparation with chlorhexidine was performed [4]. In each case, a simple or radical hysterectomy was performed alone or with other procedures as indicated by the patient's diagnosis, physical exam, and radiologic evaluation. The actual techniques used for the extrafascial type 7 total laparoscopic hysterectomy (TLH) [5], including the single field sterile skin preparation, and all surgical dissections are described elsewhere [4, 6]. No supracervical or vaginal hysterectomies were performed. A laparoscopic approach for closure of the vagina was performed in every case and is described in detail below, along with the supplementary video made in March 2014 (see Supplementary Material available online at http://dx.doi.org/10.1155/2016/1372685).

## 3. Details of Vaginal Cuff Closure Technique Incorporating Uterosacral Ligaments, Regular Placement of Sutures, and Reperitonealization

In all cases, the culdotomy was created using bipolar and monopolar electrocautery directed to a cephalad-deviated uterine manipulator cup (V-Care, ConMed, Utica, NY) to both present the cervicovaginal margin and lift that margin away from the ureters.

For culdotomy closure, two suture types were utilized. Coated braided 0-polyglactin suture on an ST-3 needle (Vicryl Endoknot, Johnson and Johnson, Cincinnati, OH, USA) was employed in the first half of the series, and monofilament 2-0 glycolide, dioxanone, and trimethylene carbonate barbed suture on a GS-22 needle (V-Loc 90, Covidien, Boulder, CO, USA) was used in the latter half. Both types of sutures have confirmed equivalent strength and absorption profiles relevant to the observation period for complications. Both sutures retain 75% of tensile strength at 14 days and are fully absorbed after a mean of 63 days (http://www.ethicon.com/healthcare-professionals/products/wound-closure/suture-assist/endoknot-suture#!description-and-specs/, http://www.covidien.com/vloc/pages.aspx?page=MaterialsGuide/). The same technique for closure was used for the duration of this report and we have reviewed all cases together.

**Figure 1:**
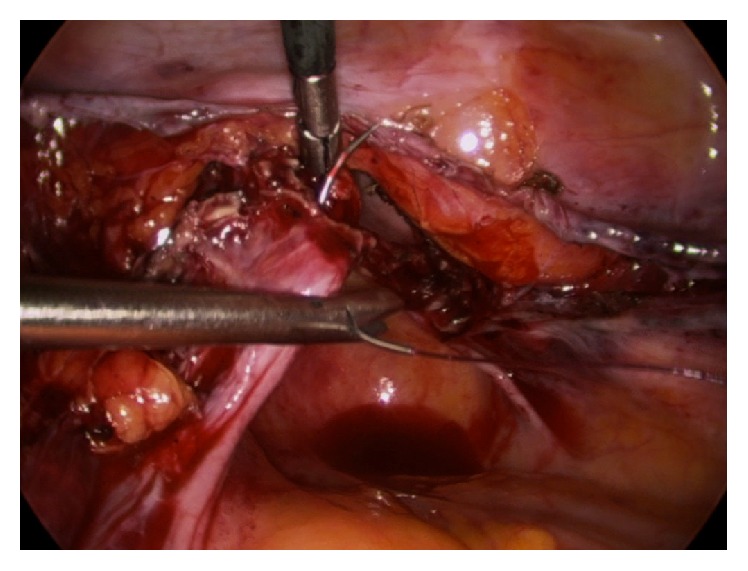
Suture is passed through the USL from about 1.5 cm posterior to the cut edge, exiting the USL fibers just proximal to the pubocervical fascia.

**Figure 2:**
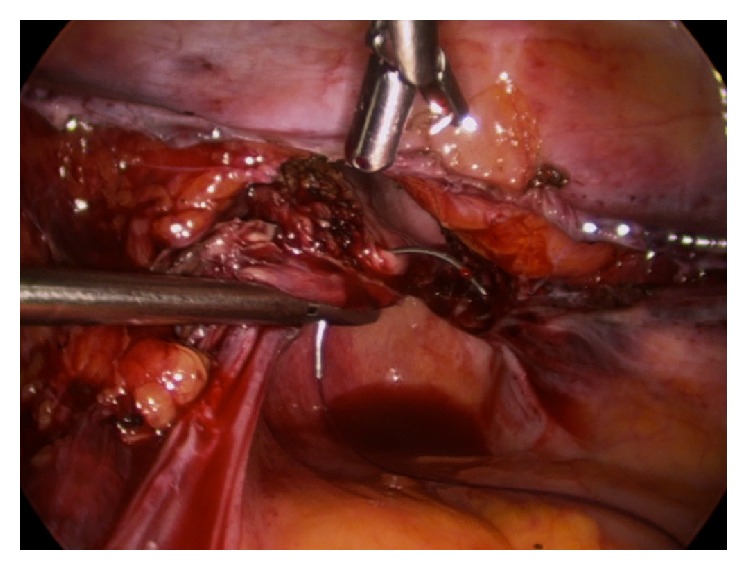
The suture is passed into the posterolateral vagina 5 mm deep to the cut edge.

**Figure 3:**
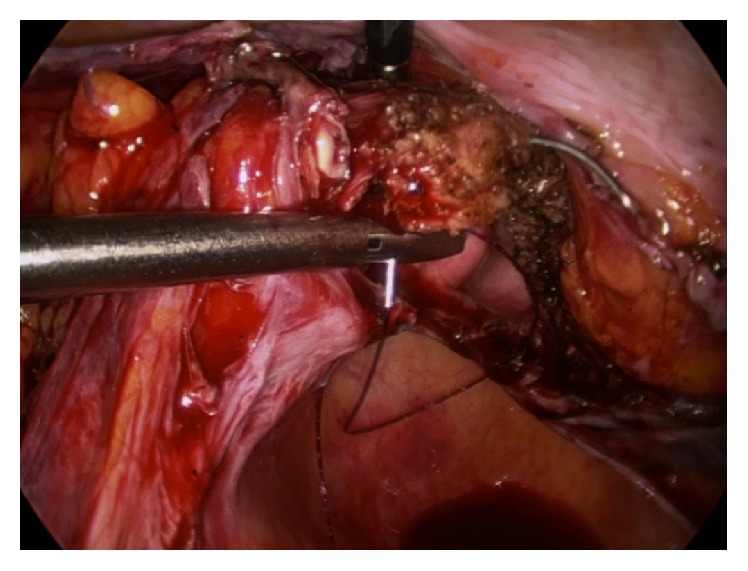
The anterolateral vaginal wall is pierced 5 mm deep from the cut edge and exits to include the cut fibers of the pubocervical fascia.

**Figure 4:**
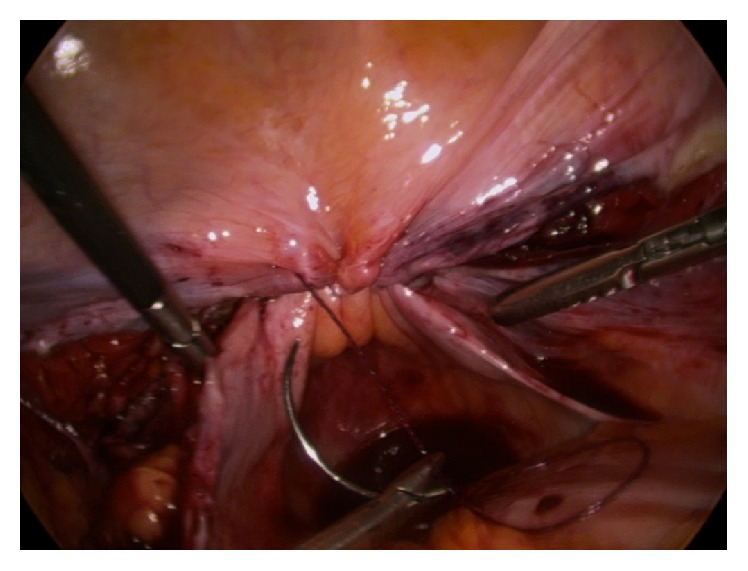
The bladder peritoneum has been sutured to the peritoneum of the anterior cul-de-sac so as to cover the raw cut edges of the vagina, while allowing drainage laterally. Note that the USLs are prominently providing support to the apex but not overly taught.

The vaginal squamous epithelial lateral-most edge (3 or 9 o'clock) is lifted anteriorly to tense and identify the uterosacral ligament (USL). The first and most lateral stitch is placed about 1.5 cm proximal to the cut edge of the USL, exiting through the posterior aspect of the transected fibers of the USL (Figure 1). Separately, it is next passed directly through the posterior vaginal cuff wall into the lumen, exiting the vaginal epithelium approximately 5 mm from the cut edge (Figure 2). The estimation of 5 mm was repeatedly based on the diameter of each of the 5 mm instruments. The suture is then passed through the lateral anterior vaginal wall, again at a depth of approximately 5 mm from the edge, exiting the tissue to be certain to include all the anterior transected fibers of the pubocervical fascia, fixing and “folding” the USL onto the posterolateral apex (Figure 3). The medial aspect of the distal uterosacral ligament is incorporated similarly into the second suture as reinforcement. Sutures are placed every 5 mm apart, at a depth of 5 mm in a noninterlocking, continuous running horizontal fashion. The contralateral USL is then incorporated in a similar fashion. The suture is run back to the contralateral side reefing through the edge of the posterior peritoneum and the anterior bladder flap peritoneum to effectively cover the cut edge of the closed vagina. This reperitonealization extends from one USL to the other but leaves the sidewalls open for drainage (Figure 4). Rarely, if there is still laxity of the USL and hypermobility of the apex, a polyester suture (Ethibond Endoknot, Johnson and Johnson, Cincinnati, OH) is used to plicate the distal USLs into the posterior cervical fascia, adding further lift to the vaginal cuff [7, 8].

Following the closure, patients were normally discharged the next day, evaluated by telephone by our nurse practitioner within two days of discharge, and contacted by the surgeon within the following week to discuss pathology results. In the absence of any report of a problem with urinary, gastrointestinal, or wound healing, the patients were seen at 6 weeks for a postoperative visit and vaginal cuff exam by either the primary author or their local gynecologist. When indicated, earlier or additional postoperative visits were scheduled.

Since there was an observational study with a significant amount of skewed data, nonparametric tests were used throughout. The Kruskal-Wallis test was used to determine if a difference existed between patients with reoperative or nonreoperative complications and patients without complications against the demographic factors of age, BMI, parity, duration of surgery, days of hospitalization, and infection. The Kruskal-Wallis is a nonparametric equivalent to the ANOVA that tests equality of distributions using rank order. Spearman's rho was used to identify any linear relationships between the demographic factors and the complications of dehiscence, bleeding, and infections. Spearman's rho is a nonparametric equivalent to the Pearson Correlation.

## 4. Results

Over the 19-year study period, 1924 patients underwent a simple or radical laparoscopic hysterectomy.  Table 1 describes the demographics of the cohort, including the preoperative diagnoses, the procedures performed, and the final pathologic diagnoses. Twenty patients had been converted to laparotomy from the planned TLH and are excluded from this analysis. Of this cohort, a total of 44 patients (2.28%) experienced a vaginal complication: 5 (.26%) had dehiscence, 13 (.68%) had bleeding, 23 (1.20%) had infection, and 3 (.16%) had adhesions (Table 2). Among the 44 patients with complications, 19 (.99%) required reoperation, while 25 (1.30%) did not require a reoperation or procedure.

There are no significant differences in demographics, operative statistics, or length of hospitalization between the patients who experienced complications and those who did not. Patients with any type of complication, reoperative or nonreoperative, were younger than those without a complication (46 versus 51 years, *p* < 0.000) and had a similar median BMI of 26.5 (range: 14.5–74.2, NS) kg/m^2^ and similar median parity of 1 (range: 0–9, NS). At surgery, the median duration of surgery was 108 minutes for all patients (range: 24–556 minutes, NS); but the median estimated blood loss for patients having a reoperative complication was higher than those without any complication (100 versus 75 cc, *p* = 0.049). While the range of days of hospitalization was less in patients with any cuff complication and with both nonreoperative and reoperative complications compared to those with no complications (1–12 versus 1–13), the Kruskal-Wallis Rank Sum Test was significant for those without any complication against reoperative patients (*p* < 0.000) indicating these two sets are distinct (Table 2).

Vaginal dehiscence was observed in five patients (0.26%), all after sexual activity, on postoperative days 30, 42, 44, 52, and 83. All of the vaginal dehiscence cases had benign pathology and simple hysterectomy. There were no differences in patient age, BMI, parity, blood loss, or hospital stay when compared with those not having dehiscence (Table 2). All of the vaginal dehiscence cases occurred in the first quarter of the study period and had utilized coated braided 0-polyglactin suture. Three patients (0.16%) had apical dehiscence larger than 2 cm, and the apical dehiscences were sutured closed; however, none of these patients had small bowel evisceration. Speculum examination of these three patients revealed only the fatty underside of the closed bladder peritoneum through the vaginal cuff defect.

Vaginal cuff bleeding occurred in 13 patients (0.68%), of whom 4 (0.21%) resolved spontaneously and 9 (0.47%) required suture repair. Patients with a bleeding complication were significantly younger that those without a bleeding complication (42 versus 52 years, *p* = 0.001) but had similar BMI, parity, surgical duration, blood loss, and length of hospital stay (Table 3). Five cases of cuff hemorrhage (0.26%) developed during the initial postoperative 23-hour hospitalization: 3 (0.16%) patients were taken back to the operating room from the recovery room, and 2 (0.10%) began bleeding in their hospital room the next morning. One patient had an unrecognized vaginal laceration from vaginal morcellation and the others from cuff arterioles. The other 4 (0.21%) reoperative cases all had bleeding from small cuff arterioles, which were electrocauterized or sutured vaginally in the office on postoperative days 4, 7, 7, and 18.

Infectious complications developed in 23 patients (1.2%). Pelvic cellulitis was clinically diagnosed in 18 patients during the office visit at 7–14 days postoperatively. All were treated with 5–7 days of oral antibiotics, typically doxycycline or ciprofloxacin, with resolution of pelvic induration and tenderness. Five patients had a CT-documented abscess, 4 (0.2%) of whom required drainage by computed tomography, while one resolved with antibiotics. While patients with infectious complications were younger than those without (47 versus 52 years, *p* = 0.011) and had a longer surgical duration (156 versus 120 minutes, *p* = 0.013) than those who did not, neither BMI, parity, blood loss, nor length of hospital stay was different (Table 2).

Small bowel obstruction from adhesions to the raw vaginal cuff was observed in 3 patients (0.2%), all of whom underwent laparoscopic lysis of adhesions on postoperative days 6-7 and recovered. All three had retracted or absent bladder peritoneum from anterior leiomyomas, a previous Cesarean section, or surgical treatment of endometriosis that precluded reperitonealization. A laparoscopic lysis of the adhesion from the cuff to the small bowel was curative in all three cases.

## 5. Discussion

The three guidelines applied to laparoscopic vaginal closure appear to be associated with an acceptably low rate of occurrence of the four major complications. The complications and concerns are discussed separately below.

### 5.1. Dehiscence Complications

With regard to dehiscence, Uccella and colleagues' systematic literature review of 13,030 patients having TLH reported 91 cases of vaginal dehiscence, with 0.64% from laparoscopic culdotomy closure, 0.18% from transvaginal closure, and 1.64% from robotic closure [9]. Hur and colleagues reviewed their hospital rates of dehiscence over ten years and reported a 1.35% dehiscence rate from TLH with evidence of a learning curve, with most of the dehiscences occurring in the first two-thirds of the period of observation [10]. With all suturing performed laparoscopically, we report a dehiscence rate of 0.26%, which compares favorably with these international rates and suggests that a standard of suture placement 5 mm deep and 5 mm interval can be learned and may be safe and possibly advantageous.

This study does not support the conclusions of Uccella et al., who attributed their low dehiscence rate by the transvaginal route to more effective knots and suture type [1]. Effective knot tying and suture reliability are key features of both of the two sutures used over the duration of the study period, resulting in excellent reliability in tissue fixation for the entire period. No dehiscences occurred in the second half of the nearly one thousand patients in whom Vicryl suture with a knot pusher was used. This fact raises doubt that suture variables and knot reliability, per se, were significant [1, 11] and points more to the surgeon's suturing skill. No study has ever reported such detail as to whether the visualized sutures at the dehisced vaginal apex had broken or whether the knots had become untied, and we did not observe those features either. In the present study, all dehiscences in our series occurred exclusively early in the* first one-quarter* of the series and resolved well before any change of suture, with no dehiscence occurrence during the latter half of the use of the first type of suture. Surgeons must introspect. We believe the dehiscence complications were due to surgeon inexperience in the early part of the series and diminished only with the surgeon's growing skill, well before the change to another suture (Figure 6). The sutures were similar enough in providing reliable fixation, for example, knot pusher and barbs with an end loop that we can only conclude that suture type is not relevant to avoidance of dehiscence but reliable and consistent suture placement is extremely relevant. A randomized trial with the two sutures would confirm or negate this.

In this series, higher blood loss correlated with risk of dehiscence but no other surgical parameters. While some studies report that patients undergoing radical hysterectomy may be at higher risk of vault dehiscence because the procedure usually shortens the vagina somewhat [12], none of the patients in this study with cervical or endometrial carcinoma undergoing radical laparoscopic hysterectomy sustained a cuff complication.

Monopolar electrosurgery use for culdotomy has been implicated by some as the cause for dehiscence [13]. However, other large series have found no impact related to the method of culdotomy incision, whether by monopolar, ultrasonic shears, cold scissors, monopolar use, or its wattage [9]. Hur and colleagues endorsed use of a low-wattage, cutting monopolar current for the culdotomy to minimize charring [10]. In the current report, bipolar sealing and a proprietary monopolar blend of cut and coagulation at 40 watts were used to create all colpotomies and to achieve cuff hemostasis. This electrocautery modality was recently reviewed by Teoh and colleagues who found that the depth of thermal injury of the culdotomy using these same instruments was only 0.6–0.7 mm [14]. While avoiding excessive charring and ineffective repetitive deep electrocautery is considered standard, this report cannot implicate monopolar current or its wattage as a factor in dehiscence.

### 5.2. Hemorrhagic Complications

A literature search did not reveal any evidence-based standards for the depth of placement or for the interval between vaginal cuff sutures. The use of 5 mm deep and 5 mm apart suture placement in this series is originally derived from the laparoscopically magnified view of the vascularity of the cuff edge. With 5 mm interval between sutures, the vessels appeared appropriately compressed and hemostatic. With a depth of 5 mm, none of the sutures pulled through the tissue, and a consistent approximation of the edge was observed when visually inspected by speculum exam after completion. Perhaps the younger patients in this study had more bleeding complications because their vaginal skin was thicker due to higher estrogen levels, possibly making adequate suturing more difficult. It is also possible that younger women had better vascularized vaginal epithelium. Suture placement consistency during each closure was achieved by comparing the diameter of any of the 5 mm instruments to gauge suture depth and interval. In theory, the ideal closure of the culdotomy results from accurate suture placement and reliable knot tying, whether vaginally or laparoscopically. Inaccurate suture placement by any route, too shallow or too far apart, can leave gaps that do not compress the small arterioles at the cuff edge or that may pull through over time or result in postoperative bleeding or dehiscence. Sutures placed too close together can cause tissue necrosis resulting in devitalized tissue that may be more susceptible to tear or dehiscence.

Surgeons relying on suture devices to reapproximate the vaginal cuff carry risk, as these can fail or be unexpectedly unavailable. Siedhoff and colleagues reported on 387 patients, all of whom had laparoscopic culdotomy closure, and found that 4.2% of patients who underwent closure with a suture device had dehiscence, while none who were closed with a barbed suture had dehiscence [11]. We observed the same absence of dehiscence in our patients closed with the barbed suture, but, as noted previously, only a randomized trial could implicate suture type and exonerate surgeon experience. Although a transvaginal route may minimize the risk of dehiscence and bleeding by affording easier and more familiar tissue handling, with potentially more precise suture placement and more reliable knots, surgeons performing laparoscopic hysterectomy should develop the basic suturing skill to close the vaginal cuff laparoscopically because variations in patient body morphology, such as high BMI, narrow vagina, and nulliparous state, may preclude a vaginal approach to cuff closure.

### 5.3. Infectious Complications

Overall, infectious complications after total abdominal or vaginal hysterectomy occur in 1.6% of patients, according to a recent report from the National Surgical Quality Improvement Program [15]. Infectious complications from laparoscopic hysterectomy were very rare in the meta-analysis by Uccella and colleagues who report an occurrence rate of 0.28%. However, these were defined in their manuscript only as “vaginal infection/abscess.” In the current series, 0.94% had cuff cellulitis, and 0.26% developed vaginal cuff abscesses. Among the risk factors reviewed in this study, duration of surgery correlated with risk of infectious complication, as others have historically described with total abdominal hysterectomy [16]. We confirm the findings of Lachiewicz and colleagues, who observed a correlation between increasing infection rate and increased* laparoscopic* operating time [3]. Surgical duration also relates to other complexities of the surgery beyond simply the hysterectomy. Young age was also found to be significantly associated with infectious complications, and this needs to be further studied.

### 5.4. Learning Curve for Culdotomy Closure Complications

Figure 6 details the occurrence of the individual categories of complications as they occurred in segments of 200 cases, demonstrating a learning curve in both the individual complication categories and in the total rate of complications. While the standards were set early in the series, it appears that surgeon experience affects the success of the applications of the guidelines. Comparing overall complications in this series with other series reveals this rate is favorable. There were no fatalities in this series as some have observed in the early years after the procedure was described [17]. However, surgeons in the 21st century now have the widely available benefit of training in advanced laparoscopic procedures in both residency and postgraduate continuing medical education courses which were not available when this series started. Learning curves now should not be as steep as they were in the 1990s [18].

### 5.5. Concerns about Support

It is estimated that United States women have a lifetime risk of 11% for prolapse surgery or 200,000 surgeries yearly for prolapse or incontinence [19, 20].

**Figure 5:**
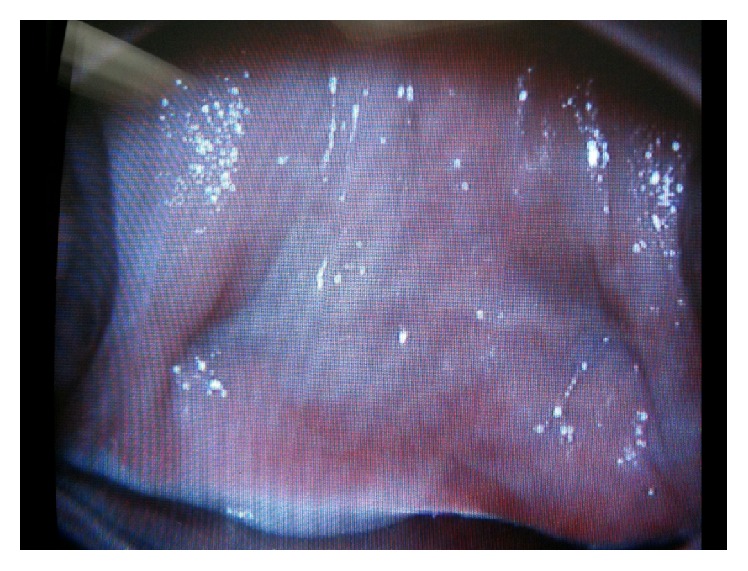
In this speculum exam photo from the six-week postoperative office check for granulation, good lateral apical support is seen bilaterally from the dimple caused by the uterosacral ligaments.

**Figure 6:**
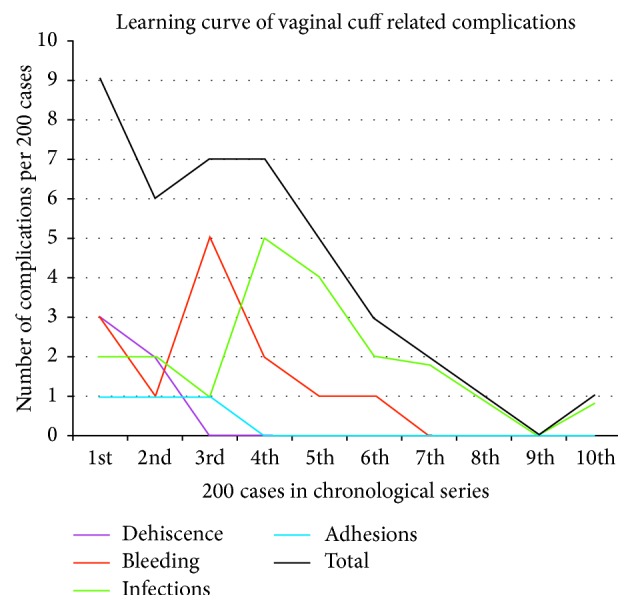
This chart reflects the complications separated in segments of 200 cases each, over the 19 years of this study. Dehiscence (purple), bleeding (red), infections (green), and adhesions (blue) show gradual decrease over time. The total decrease in complications (black) is due to a variety of factors including the surgeons' learning curve, improved surgical skills, experience, and judgment.

Surgeons have been urged to assess prolapse and continence prior to every hysterectomy and to try to prevent need for a future prolapse surgery [20]. Since 2008, it has been accepted that securing the uterosacral ligaments bilaterally to the pubocervical fascia restores and enhances level I support and decreases apical vault prolapse and may prevent subsequent prolapse surgery [21]. The baseline data from the Women's Health Initiative (WHI) of 10,727 women who had a hysterectomy by any approach, compared with 16,616 women retaining their uterus, showed very similar rates of stress incontinence (24% versus 26%), as well as cystocele (33% versus 34%) and rectocele (18% versus 19%) [22, 23]. Only parity and obesity affected prolapse in the total WHI population, not hysterectomy. While these findings offer reassurance that hysterectomy in itself does not cause prolapse or incontinence, they also suggest that we, as surgeons, are not addressing mild to moderate support issues when we perform hysterectomy for non-prolapse-related gynecologic indications [24]. Our reattachment of the uterosacral ligament replicates the first portion of the entire technique of colposuspension and at least appears to maintain and slightly enhance level 1 support. Laparoscopic approaches may provide a better opportunity for prolapse repair given that the anatomy is so well visualized. Laparoscopic uterosacral ligament suspension was shown to provide better apical support and fewer reoperations for prolapse than vaginal approach for the same procedure (Figure 5) [25, 26]. It may be that the approach from within the peritoneal cavity allows surgeons to more confidently identify the uterosacral ligament and provide better level I support [16]. Although long-term follow-up of vaginal support issues for all patients referred from afar to this oncology-based practice is not part of this report, we can confirm that there have been six returns to our practice for prolapse issues out of 1,924. All patients were instructed to report all complications to our office for ongoing care and management, but all patients were not surveyed or brought back for exam, resulting in a possible undercount of our complications. Quality of follow-up is a major weakness of this retrospective report, and further study is needed.

### 5.6. Closing the Bladder Peritoneum

The technique of closing the “bladder flap,” or reperitonealization of the vaginal cuff, after total abdominal hysterectomy was largely abandoned after two retrospective case cohort reports from 1983 and 1994 [27, 28]. The authors hypothesized that the peritoneum regrows quickly over the denuded pelvic tissues and that the sutures may contribute to subsequent adhesion formation. However, in a recent prospective trial of laparotomy closure in rabbits, closure of the peritoneum, regardless of suture type, reduced the amount of adhesions seen at reoperation 14 days later [29]. Parietal peritoneal closure after Cesarean section has been shown to cause fewer dense or filmy adhesions [30].

In some instances, due to anatomic limitations, the peritoneum could not be reapproximated, but the frequency of this was not recorded. This is especially true for patients with large, anterior leiomyomas, extensive endometriosis, or multiple prior Cesarean sections, which disrupt the bladder peritoneum and/or make it less pliant or adherent to the uterotomy site. The three patients in this series who developed small bowel adhesions to the raw vaginal apex all had anatomy that precluded covering the raw vaginal edges with peritoneum. While small bowel adhesions have been reported from the use of the polydioxanone barbed suture [31], in this study, none of the patients who had reperitonealization experienced a complication from adhesions.

In this series, three of the five patients with dehiscence required suture closure of the apex for a rupture larger than 2 cm, but none experienced intestinal evisceration. A literature review by Hur and colleagues in 2007 found evidence that from 30 to 95% of dehiscences were associated with evisceration [10]. While Uccella and colleagues found that peritoneal closure did not reduce dehiscence, there was no stratification of data by need for* reoperation for perforation* based on peritoneal closure over the culdotomy closure [9]. Potentially, closing the pliant bladder peritoneal layer over the raw vaginal cuff edge offers one additional layer of protection from catastrophic bowel evisceration for those few who do experience disruption of the vaginal cuff. The fact that the speculum exam in the emergency room showed the intact underside of the peritoneal layer in two of our cases of dehiscence motivates us to continue this practice until prospective studies provide more information.

There are several weaknesses in this retrospective report. First, some of the patients, living as far as nine hours away, may have experienced cuff complications and presented at another hospital for their emergency care. While all patients receive printed material describing when and how to contact the surgeon in the event of suspected complications, it is possible that they obtained local care without our knowledge, resulting in underestimation of the complication rate. The fact that one surgeon performed all the surgeries may reduce the likelihood of broader reproducibility; however, the skillset required in TLH is not beyond the certified gynecologist. Surgeon learning curve over 19 years changes in surgical practice, and specific experience with the two sutures precludes any specific conclusion about the sutures over the study period. Tissue healing is also dependent on many factors not specifically addressed herein, including comorbid medical conditions (diabetes mellitus, hypertension), steroid use, nutritional status, general health, and compliance with postoperative instructions. There was also no formal pre- or postoperative formal POP-Q assessment of level 1 support to confirm effectiveness of USL incorporation procedures.

## 6. Conclusions

All vaginal closures should be individualized to optimize support and avoid dehiscence, bleeding, infection, and adhesions. The low rate of the four major types of complications using these laparoscopic culdotomy closure guidelines suggests that these guidelines are safe. Our belief is that the suture material made no difference in the low rate of complications after the first-quarter of the series was completed. Rather, surgeon accuracy in suture placement with a depth of 5 mm and 5 mm interval, uterosacral ligament incorporation for apical support, and reperitonealization where possible appear most important; but a large, prospective clinical trial is needed to delineate the safest standards for laparoscopic culdotomy closure and to show which portion(s) of this technique contribute most significantly to a low rate of complications. Additionally, a large, long-term prospective analysis would be necessary to confirm any level 1 support benefit from the reattachment of the uterosacral ligament to the pubocervical fascia during the vaginal cuff closure.

## Supplementary Material

In this brief video, the uterosacral ligament is sutured to the pubocervical fascia, each stitch is placed 5mm apart and 5mm deep to the vaginal cuff edge, and then, whenever possible, the bladder flap is sutured over the cut edge of the cuff.

## Figures and Tables

**Table 1:** Demographics of all 1924 patients in study.

Feature	Mean	Range

Age (years)	52	(15–97)
BMI (Kg/m^2^)	29	(14–74)
Parity (#)	1	(0–9)
Duration of surgery (minutes)	121	(24–556)
Uterine weight (Kg)	216	(14–3131)
Pelvic nodes (#)	23	(0–61)
Inframesenteric nodes (#)	7	(0–38)
Infrarenal nodes (#)	7	(0–37)
Transfusions (#PRBC)	0	(0–5)
Hospital stay (days)	1	(1–13)

Preoperative diagnosis	*N*	(%)

Pelvic mass	415	21.6%
Leiomyoma	400	20.8%
Endometrial carcinoma	355	18.5%
Pelvic pain	126	6.5%
BrCa+/family history	108	5.6%
Endometrial hyperplasia	100	5.2%
Transgender	94	4.9%
Adenomyosis	86	4.5%
Prolapse	68	3.5%
Cervical carcinoma	49	2.5%
Ovarian/tubal carcinoma	35	1.8%
Cervical dysplasia	31	1.6%
Menorrhagia	25	1.3%
Stress incontinence	15	0.8%
Uterine stromal/other	13	0.7%
Menstrual migraines/PCOS	3	0.2%
PID	1	0.1%

Procedure	*N*	%

TLH-BSO	1723	90%
Appendectomy	1103	57%
Any lymphadenectomy	205	11%
McCall's colposuspension	149	8%
Adhesiolysis	134	7%
Endometriosis resection	83	4%
Burch	77	4%
RLH-BSO	76	4%
TLH	68	4%
TLH-BS	48	3%
TOT	26	1%
Ureterolysis	21	1%
Herniorrhaphy	13	1%
Posterior repair	11	1%
Minilaparotomy	7	0%
Cholecystectomy	6	0%
Converted	5	0%
Tumor debulking	5	0%
RLH-BS	4	0%
Converted	3	0%
Episiotomy	2	0%

Postoperative diagnosis	*N*	%

Leiomyoma	615	32.0%
Endometrial carcinoma	368	19.1%
Benign ovarian neoplasia	363	18.9%
Adenomyosis	174	9.0%
Endometriosis	127	6.6%
Ovarian/tubal carcinoma	98	5.1%
Endometrial hyperplasia	71	3.7%
Cervical carcinoma	49	2.5%
Cervical dysplasia	26	1.4%
Adhesions	15	0.8%
Uterine stromal/other	9	0.5%
Metastatic cancer	7	0.4%
PID	2	0.1%

**Table 2:** Comparing demographic factors of those without a cuff complication with those having any or nonreoperative or reoperative complications.

Demographics	No cuff complication	*All* types of cuff complications	
	*N* = 1880 (97.7)	*N* = 44 (2.2%)	
	Median (range)	Median (range)	*p* value
Age (years)	51 (15–97)	46 (25–75)	0
BMI (kg/m^2^)	26.5 (14.5–74.2)	25.1 (18.2–49.1)	0.078
Parity (#)	1 (0–9)	1 (0–9)	0.94
Surgery duration (min)	108 (240–556)	121 (45–305)	0.118
Estimated blood loss (mL)	75 (24–556)	100 (5–1000)	0.06
Hospital stay (days)	1 (1–13)	1 (1–12)	0.025

Demographics	No cuff complication	*Nonreoperative* complications	
	*N* = 1880 (97.7)	*N* = 25 (1.3%)	
	Median (range)	Median (range)	*p* value

Age (years)	51 (15–97)	44 (27–75)	0.003
BMI (kg/m^2^)	26.5 (14.5–74.2)	25.2 (19.1–49.1)	0.167
Parity (#)	1 (0–9)	1 (0–4)	0.359
Surgery duration (min)	108 (240–556)	119 (45–305)	0.446
Estimated blood loss (mL)	75 (24–556)	100 (5–400)	0.443
Hospital stay (days)	1 (1–13)	1 (1-2)	0.785

Demographics	No cuff complication	*Reoperative* cuff complications	
	*N* = 1880 (97.7)	*N* = 19 (.9%)	
	Median (range)	Median (range)	*p* value

Age (years)	51 (15–97)	46 (24–58)	0.007
BMI (kg/m^2^)	26.5 (14.5–74.2)	24.8 (18.2–42)	0.28
Parity (#)	1 (0–9)	2 (0–9)	0.349
Surgery duration (min)	108 (240–556)	130 (50–285)	0.137
Estimated blood loss (mL)	75 (24–556)	100 (10–1000)	0.049
Hospital stay (days)	1 (1–13)	1 (1–12)	0

**Table 3:** Relationship between demographic factors and complications.

Factor	Dehiscence		Bleeding		Infections	
	*N* = 5 (0.3%)		*N* = 13 (.68%)		*N* = 23 (1.2%)	
	95% CI	*p* value	95% CI	*p* value	95% CI	*p* value
Age (years)	40–58	0.297	33–49	0.001	43–49	0.011
BMI (kg/m^2^)	15.0–27.5	0.113	12.6–30.2	0.194	23.6–28.7	0.726
Parity (#)	1,3	0.31	50–135	0.618	0-1	0.82
Surgery duration (min)	80–180	0.732	10–200	0.175	104–195	0.013
Estimated blood loss (mL)	5–300	0.683	1,2	0.687	100–200	0.039
Hospital stay (days)	1,2	0.1	0-1	0.35	1,1	0.637
